# Supplementary material for: The effects of drought and nutrient addition on soil organisms vary across taxonomic groups, but are constant across seasons
Source: Sci Rep. 2019 Jan 24;9:639. doi: 10.1038/s41598-018-36777-3 (PMC6345851; doi:10.1038/s41598-018-36777-3)
Supplement: Supplementary file 1 — Supplementary Information [file 41598_2018_36777_MOESM1_ESM.docx]

# Supplementary Information

Manuscript: The effects of drought and nutrient addition on soil organisms vary across taxonomic groups, but are constant across seasons

Authors: Julia Siebert*, Marie Sünnemann, Harald Auge, Sigrid Berger, Simone Cesarz, Marcel Ciobanu, Nathaly R. Guerrero-Ramírez, Nico Eisenhauer

*corresponding author (julia.siebert@idiv.de)


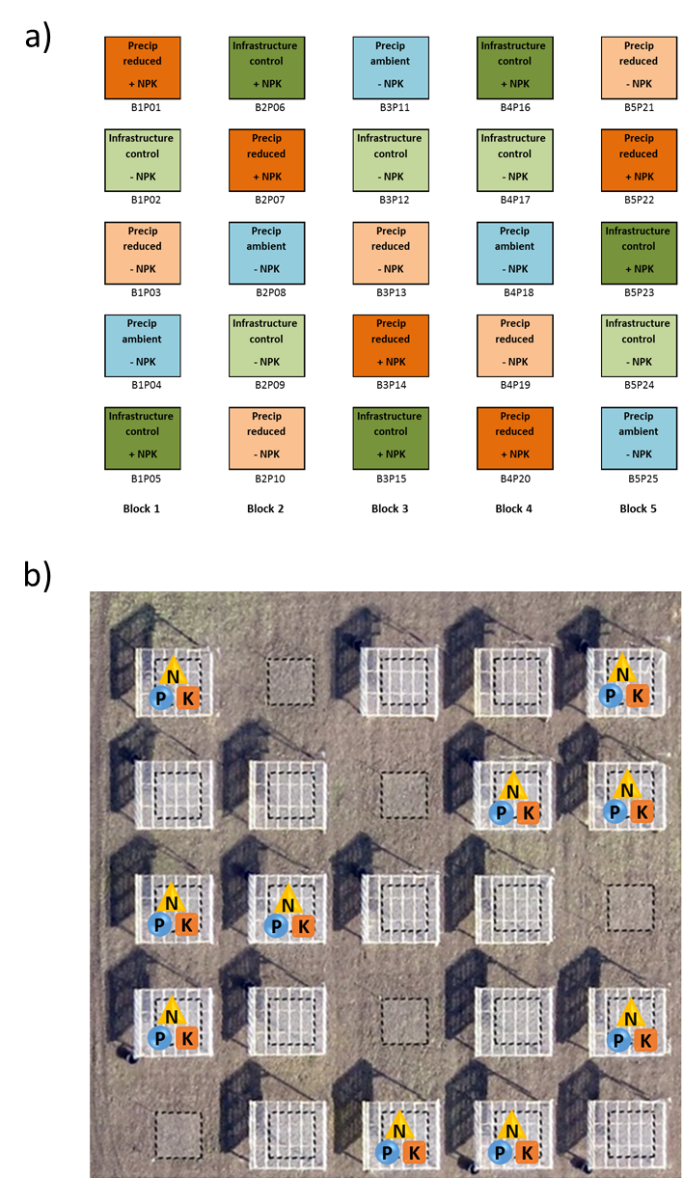


**Figure S1. The experimental design of the Drought-Net experiment in Bad Lauchstädt, Germany.** (a) The experiment consists of 25 plots (2 x 2 m) arranged in five blocks. The two manipulation factors drought and fertilization are fully crossed and randomly arranged within the blocks. (control = light green, drought = light orange; NPK fertilization = green; and drought x NPK fertilization = orange). In addition, one plot per block receives ambient precipitation without any roof construction to control for effects of the roof construction itself (blue, not crossed with fertilization). (b) Aerial picture of the experimental site with indication of the fertilization treatment.


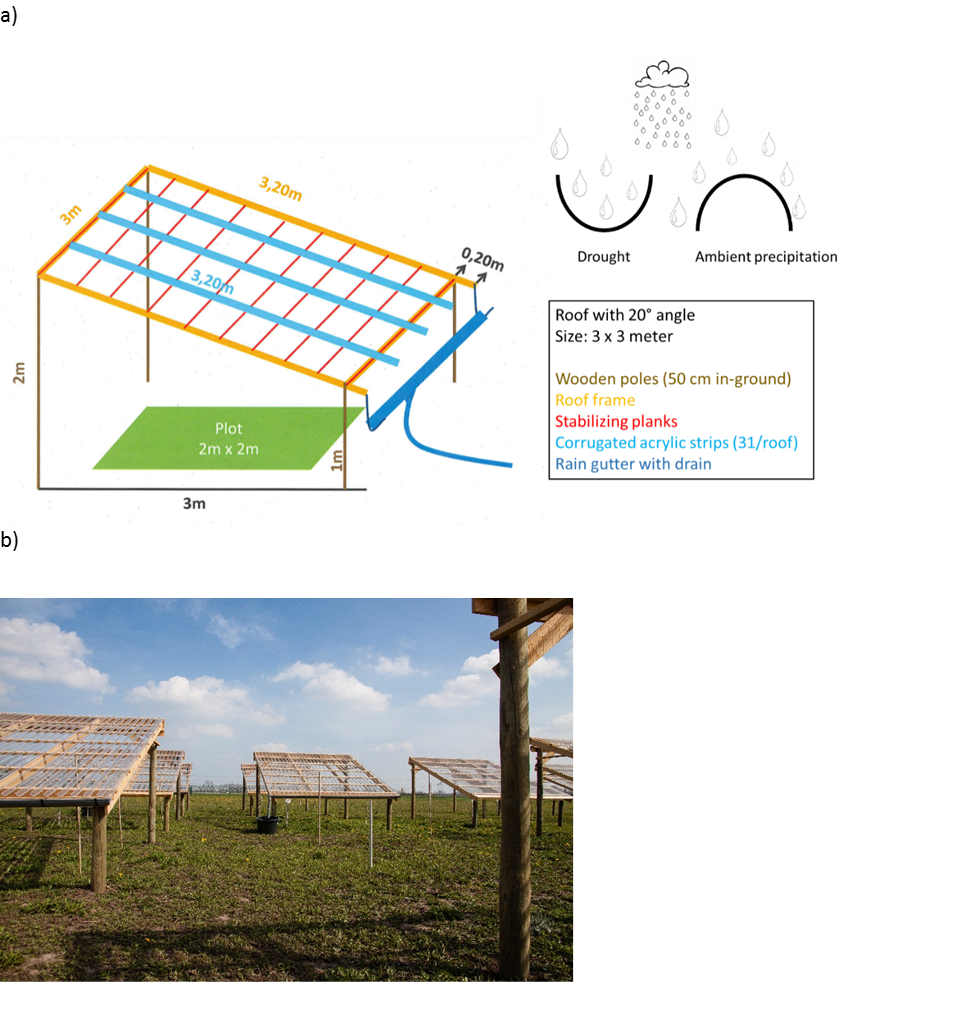


**Figure S2. The roof construction of the precipitation treatment.** (a) For the drought treatment the corrugated acrylic strips are mounted in a convex way, for the infrastructure control the acrylic strips are mounted in a concave way not keeping rainwater off from falling on the plot. Conceptual drawings by J. Siebert & S. Berger. (b) The realized roof constructions of the Drought-Net site in Bad Lauchstädt, Germany. Photo copyright: Julia Siebert.

**
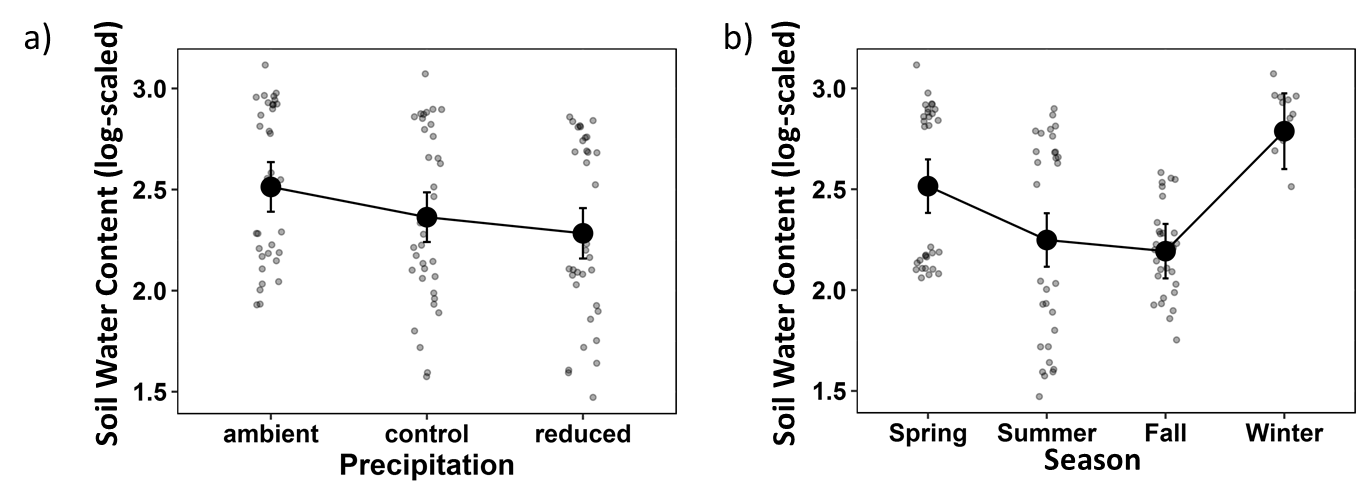
**

**Figure S3. The effects of the precipitation treatment on soil water content (%) based on mixed effects model fits.** (a) Across all eight sampling campaigns; and (b) separated by season. Ambient = ambient precipitation (without roof construction); control = infrastructure control (roof construction with concave acrylic strips); and reduced = drought (roof constructions with convex acrylic strips). Error bars indicate 95% confidence intervals.


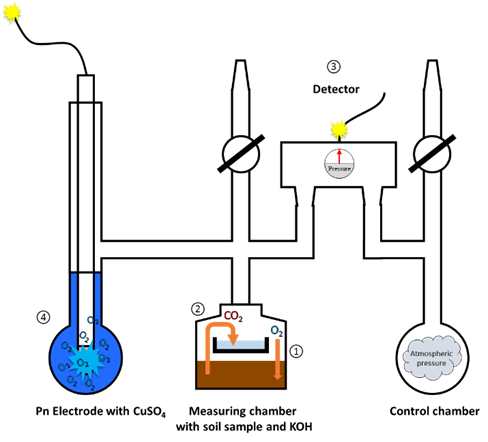


**Figure S4. The O_2_-microcompensation system (Scheu 1992).** (1) Microorganisms in the soil sample consume O_2_; (2) the released CO_2_ is immediately absorbed by KOH in the chamber of the sample, thus leading to a difference in the pressure of the measuring chamber and the control chamber (which are operating in a closed system at constant temperature); (3) the pressure difference is noticed by a detector; and (4) an electrolytic reaction is initiated (one electric pulse) that provides a specific amount of O_2_ to equalize the pressure between the chambers. The microbial respiration determined by this method is based on the amount of detected pulses per time, which are afterwards recalculated to consumed O_2_ in the soil sample.


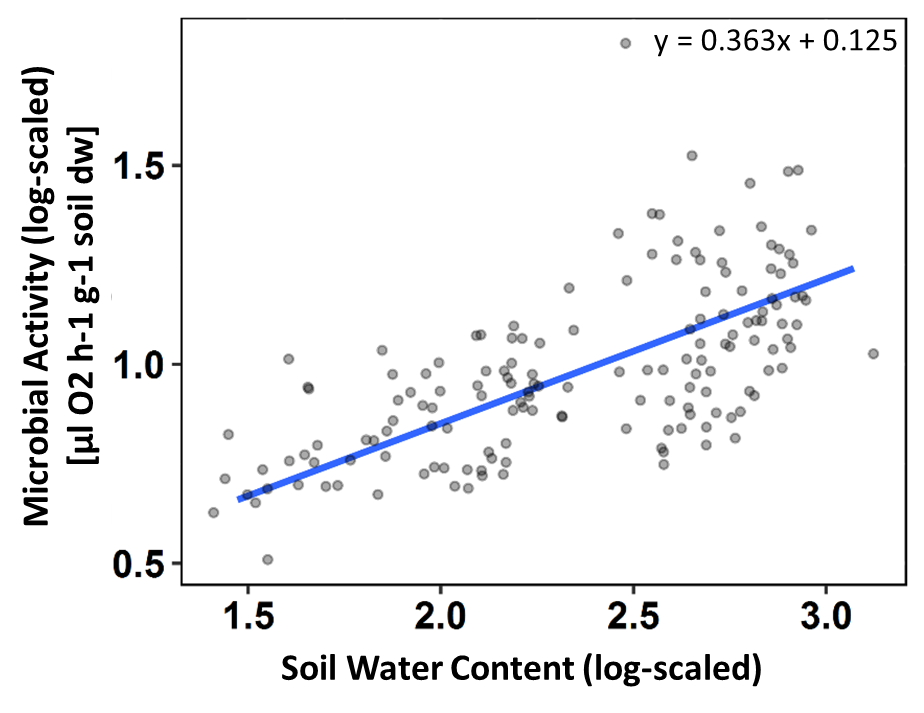


**Figure S5. Effect of soil water content on soil microbial activity (both log-scaled) based on mixed-effects model fits across all seasons (R^2^ (marginal) = 0.42; R^2^ (conditional) = 0.68 of the full model, see Table S5).**


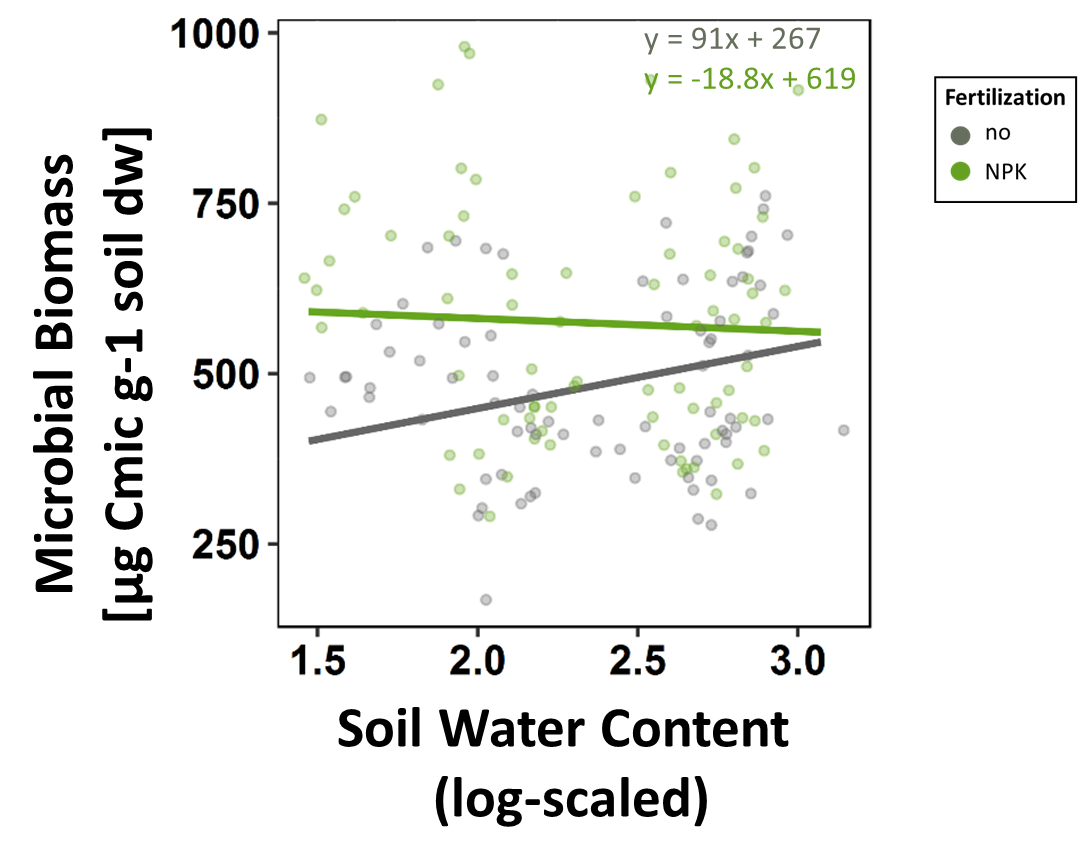


**Figure S6. Effects of soil water content (log-scaled) on soil microbial biomass dependent on the NPK fertilization treatment.** Lines are mixed-effects model fits with and without fertilization across all seasons (R^2^ (marginal) = 0.07; R^2^ (conditional) = 0.82 of the full model, see Table S6). Grey = no NPK fertilization, green = NPK fertilization.


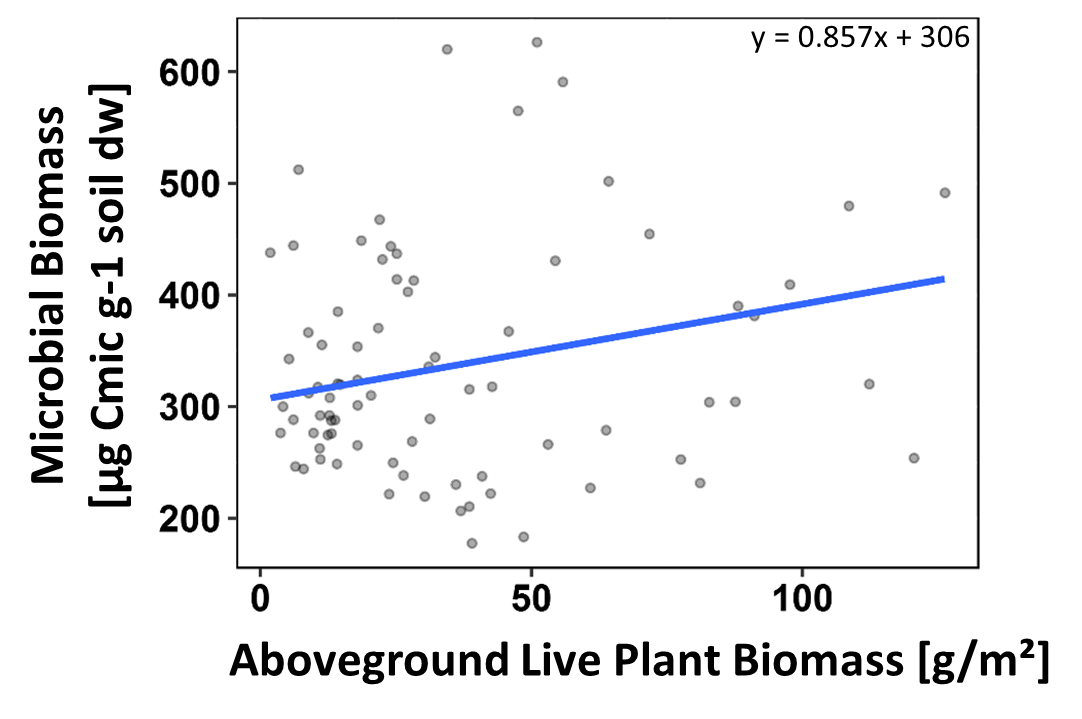


**Figure S7. Effects of plant biomass on soil microbial biomass based on mixed effects model fits (R^2^ (marginal) = 0.05; R^2^ (conditional) = 0.68, see Table S7).** Four sampling campaigns from June and September 2016 as well as 2017 were included.


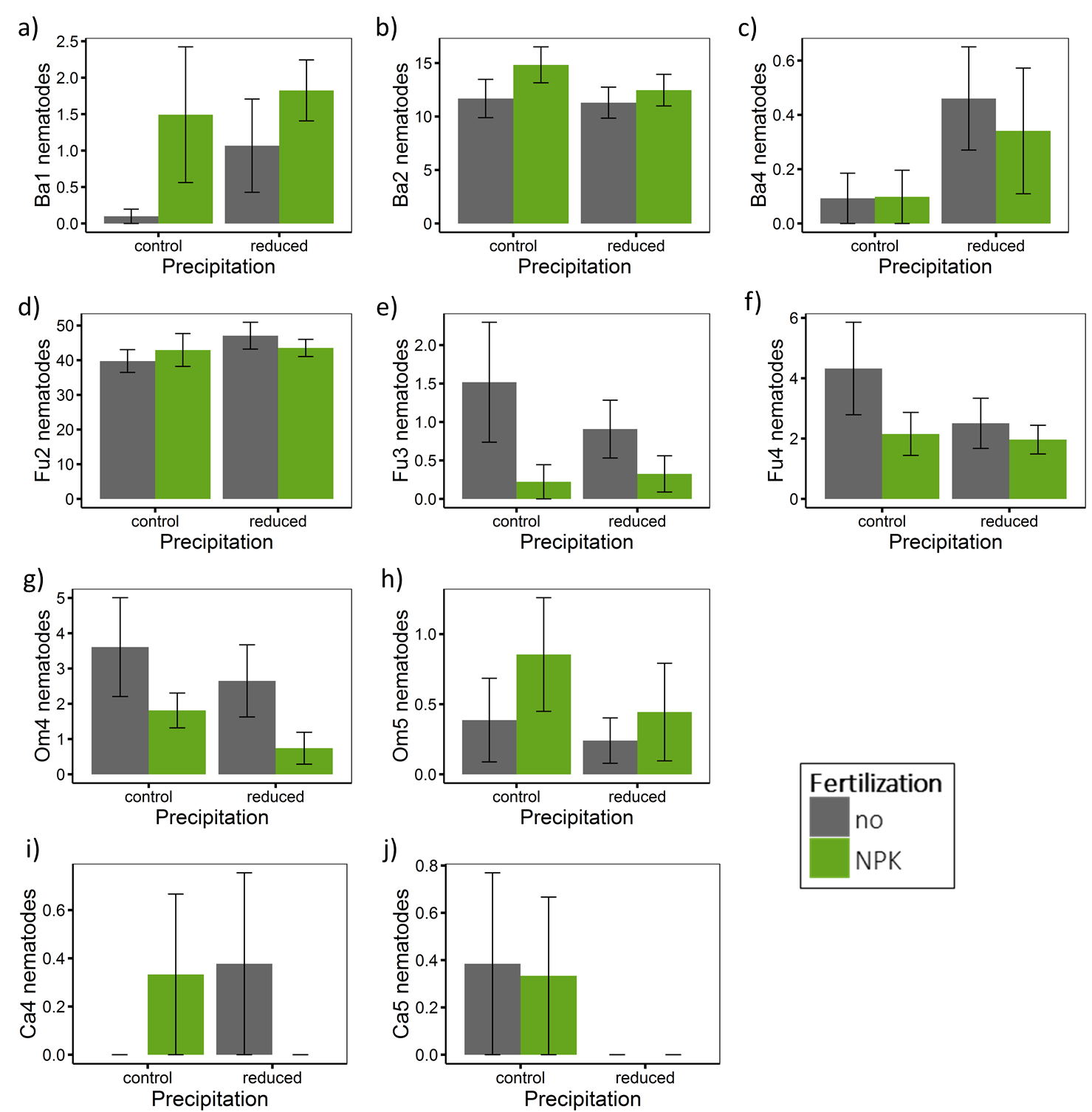


**Figure S8.** Barplots showing the effects of drought and fertilization on nematodes belonging to the following functional guilds based on proportionate shares in the community (a) Ba_1_; (b) Ba_2_; (c) Ba_4_; (d) Fu_2_; (e) Fu_3_; (f) Fu_4_; (g) Om_4_; (h) Om_5_; (i) Ca_4_; and (j) Ca_5_^49,50^. Means ± SE. Grey = no NPK fertilization; green = NPK fertilization. Two seasons (spring and summer 2017) were included.


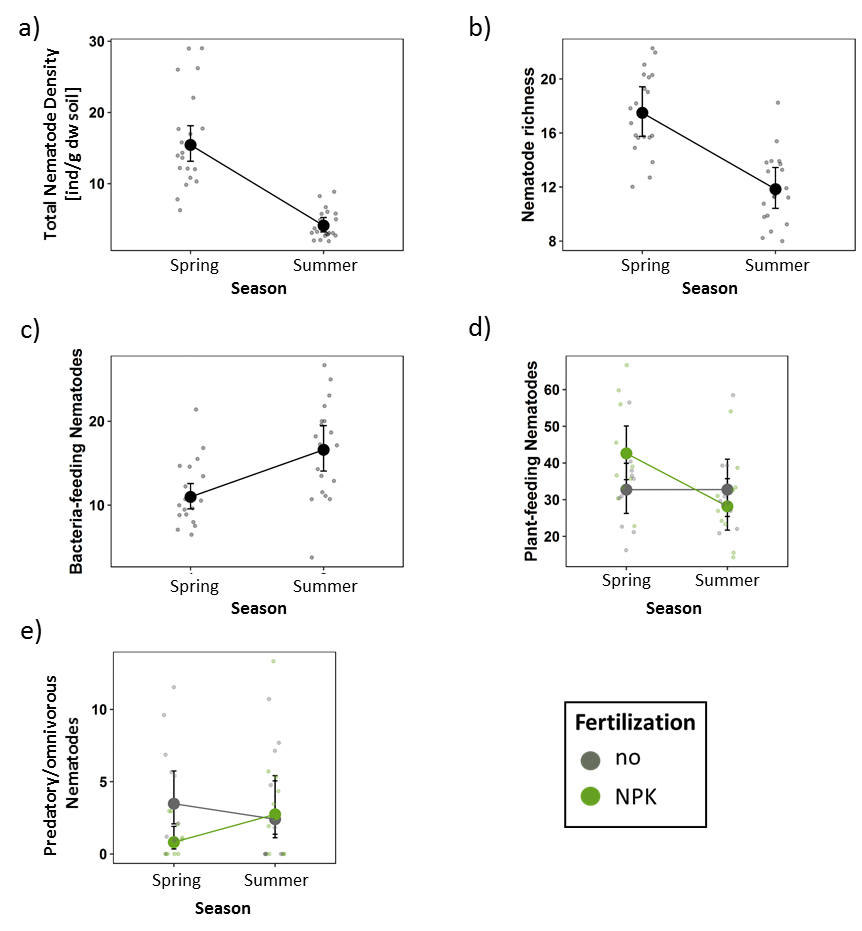


**Figure S9.** The effects of season (spring and summer 2017) on (a) nematode density, (b) richness, and the percentages of (c) bacteria-feeding nematodes, (d) plant-feeding nematodes (interacting with NPK fertilization), and (e) predatory/omnivorous nematodes (interacting with NPK fertilization) based on mixed effects model fits. Error bars indicate 95% confidence intervals. Grey = no NPK fertilization; green = NPK fertilization.


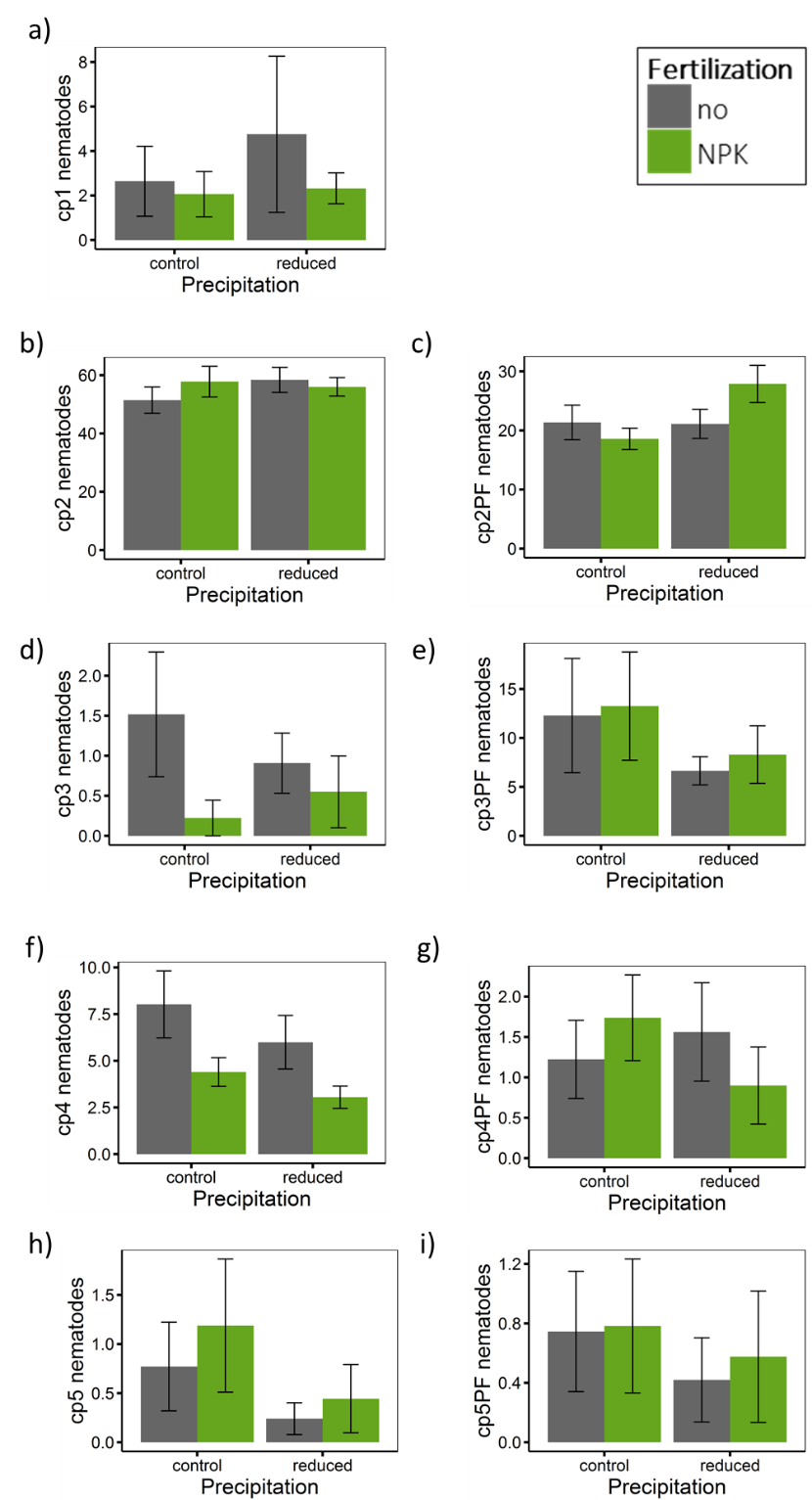


**Figure S10.** Barplots showing the effects of drought and fertilization on nematodes belonging to the following cp categories based on proportionate shares in the community (a) cp1; (b) cp2; (c) cp2PF; (d) cp3; (e) cp3PF; (f) cp4; (g) cp4PF; (h) cp5; and (i) cp5PF (PF = plant feeders)^49,50^. Means ± SE. Grey = no NPK fertilization; green = NPK fertilization. Two seasons (spring and summer 2017) were included.


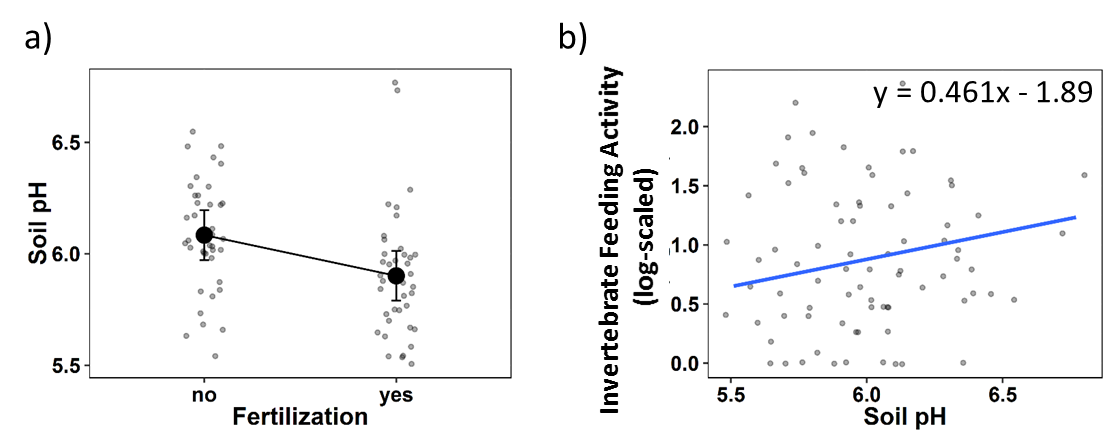


**Figure S11. Soil pH.** (a) The effects of NPK fertilization on soil pH based on mixed effects model fits (R^2^ (marginal) = 0.11; R^2^ (conditional) = 0.53 of the full model, see Table S7). Error bars indicate 95% confidence intervals. (b) Effects of soil pH on invertebrate feeding activity (log-scaled) based on mixed effects model fits (R^2^ (marginal) = 0.04; R^2^ (conditional) = 0.41, see Table S8). Data from spring, summer, fall, and winter 2017 is included.

**Table S1. Effects of the precipitation treatment (ambient, infrastructure control, and drought) and season on soil water content based on linear mixed-effects models (R² marginal = 0.30, R² conditional = 0.37).** A random intercept with plots nested within blocks, was added to the model and a compound symmetry covariance structure was used to account for repeated measurements within plots. Significant results are shown in bold.

| Treatment | *F-value* | *p-value* |
| --- | --- | --- |
| Precipitation  Season  Precipitation x Season | 45.20  9.49  0.35 | **< 0.001**  **< 0.001**  0.90 |

**Table S2. Mean values of all soil response variables.** Shown are means of the non-transformed data (±SE) for the different treatment combinations (control, NPK fertilization, drought, drought x NPK fertilization).

|  |  | | | | | | | |
| --- | --- | --- | --- | --- | --- | --- | --- | --- |
|  | **Control** | | **NPK** | | **Drought** | | **Drought x NPK** | |
| **Soil response variable** | *Mean* | ±*SE* | *Mean* | ±*SE* | *Mean* | ±*SE* | *Mean* | ±*SE* |
| **Soil invertebrate feeding activity** | 3.14 | 0.35 | 1.36 | 0.197 | 1.52 | 0.26 | 1.14 | 0.22 |
| **Microbial activity** | 1.65 | 0.08 | 1.76 | 0.10 | 1.76 | 0.09 | 1.81 | 0.09 |
| **Microbial biomass** | 469.08 | 19.71 | 568.88 | 27.26 | 499.68 | 21.49 | 584.86 | 27.66 |
| **Nematodes per g dw soil** | 10.10 | 2.54 | 11.34 | 2.52 | 7.899 | 1.76 | 11.68 | 3.08 |
| **Nematode richness** | 14.3 | 1.3 | 15.5 | 1.38 | 14.3 | 1.29 | 14.6 | 1.15 |
| **Enrichment Index** | 43.94 | 0.80 | 45.16 | 2.22 | 46.37 | 0.90 | 47.61 | 0.90 |
| **Structure Index** | 41.10 | 6.00 | 28.57 | 4.37 | 30.07 | 6.51 | 21.46 | 3.82 |
| **Channel Index** | 99.29 | 0.71 | 90.42 | 5.52 | 93.39 | 3.82 | 86.05 | 3.08 |
| **Maturity Index** | 2.34 | 0.07 | 2.17 | 0.04 | 2.21 | 0.06 | 2.10 | 0.03 |
| **Plant-feeding nematodes** | 35.60 | 4.10 | 34.35 | 5.50 | 29.73 | 2.56 | 37.64 | 3.48 |
| **Fungal-feeding nematodes** | 45.60 | 2.59 | 45.33 | 4.94 | 50.49 | 3.53 | 45.82 | 2.38 |
| **Bacteria-feeding nematodes** | 11.87 | 1.74 | 16.42 | 1.53 | 12.82 | 1.89 | 14.86 | 1.54 |
| **Omnivorous and predatory nematodes** | 4.38 | 1.46 | 3.33 | 1.29 | 3.27 | 1.07 | 1.18 | 0.50 |
| **Ba_1_-nematodes** | 0.10 | 0.10 | 1.49 | 0.93 | 1.07 | 0.64 | 1.83 | 0.42 |
| **Ba_2_-nematodes** | 11.68 | 1.79 | 14.83 | 1.68 | 11.30 | 1.45 | 12.47 | 1.48 |
| **Ba_4_-nematodes** | 0.09 | 0.09 | 0.10 | 0.10 | 0.46 | 0.19 | 0.34 | 0.23 |
| **Fu_2_-nematodes** | 39.76 | 3.28 | 42.95 | 4.74 | 47.08 | 3.87 | 43.53 | 2.48 |
| **Fu_3_-nematodes** | 1.52 | 0.78 | 0.22 | 0.22 | 0.91 | 0.38 | 0.32 | 0.24 |
| **Fu_4_-nematodes** | 4.32 | 1.53 | 2.15 | 0.71 | 2.51 | 0.83 | 1.97 | 0.48 |
| **Om_4_-nematodes** | 3.61 | 1.40 | 1.81 | 0.49 | 2.65 | 1.02 | 0.74 | 0.45 |
| **Om_5_-nematodes** | 0.39 | 0.30 | 0.85 | 0.41 | 0.24 | 0.16 | 0.44 | 0.35 |
| **Ca_4_-nematodes** | 0 | 0 | 0.33 | 0.33 | 0.38 | 0.38 | 0 | 0 |
| **Ca_5_-nematodes** | 0.38 | 0.38 | 0.33 | 0.33 | 0 | 0 | 0 | 0 |
| **cp1 nematodes** | 2.64 | 1.57 | 2.06 | 1.01 | 4.75 | 3.51 | 2.32 | 0.69 |
| **cp2 nematodes** | 51.45 | 4.49 | 57.79 | 5.29 | 58.37 | 4.32 | 56.00 | 3.17 |
| **cp2 plant-feeding nematodes** | 21.35 | 2.93 | 18.57 | 1.81 | 21.11 | 2.47 | 27.86 | 3.15 |
| **cp3 nematodes** | 1.52 | 0.78 | 0.22 | 0.22 | 0.91 | 0.38 | 0.55 | 0.45 |
| **cp3 plant-feeding nematodes** | 12.28 | 5.84 | 13.25 | 5.53 | 6.64 | 1.44 | 8.30 | 2.94 |
| **cp4 nematodes** | 8.02 | 1.79 | 4.40 | 0.77 | 5.99 | 1.43 | 3.05 | 0.60 |
| **cp4 plant-feeding nematodes** | 1.22 | 0.48 | 1.74 | 0.53 | 1.56 | 0.61 | 0.90 | 0.48 |
| **cp5 nematodes** | 0.77 | 0.45 | 1.19 | 0.68 | 0.24 | 0.16 | 0.44 | 0.35 |
| **cp5 plant-feeding nematodes** | 0.75 | 0.40 | 0.78 | 0.45 | 0.42 | 0.28 | 0.58 | 0.44 |

**Table S3. Effects of drought, fertilization, soil water content (log-scaled) and their interactions on the activity of soil microbes (log-scaled basal respiration) based on linear mixed effects models (R² marginal = 0.42, R² conditional = 0.68).** A random intercept with plots nested within blocks, which were nested within year was added to the model. A compound symmetry covariance structure was used to account for repeated measurements within plots. Significant results are shown in bold.

| Treatment | F-value | p-value |
| --- | --- | --- |
| Drought  Fertilization  Soil water content  Drought x Fertilization  Drought x Soil water content  Fertilization x Soil water content  Drought x Fertilization x Soil water content | 0.48  4.04  170.83  0.006  0.006  0.004  0.054 | 0.49  0.05  **< 0.001**  0.94  0.94  0.95  0.82 |

**Table S4. Effects of drought, fertilization and soil water content on microbial biomass based on linear mixed effects models (R² marginal = 0.07, R² conditional = 0.82).** A random intercept with plots nested within blocks, which were nested within year was added to the model. A compound symmetry covariance structure was used to account for repeated measurements within plots. Significant results are shown in bold.

| Treatment | F-value | p-value |
| --- | --- | --- |
| Drought  Fertilization  Soil water content  Drought x Fertilization  Drought x Soil water content  Fertilization x Soil water content  Drought x Fertilization x Soil water content | 1.82  36.88  4.64  0.19  0.91  10.60  0.000 | 0.19  **< 0.001**  **0.03**  0.66  0.34  **0.002**  0.98 |

**Table S5. Effects of plant biomass on soil microbial biomass based on linear mixed-effects models (R² marginal = 0.05, R² conditional = 0.68).** A random intercept with blocks nested within samplings was added to the model and a compound symmetry covariance structure was used to account for repeated measurements within plots. Significant results are shown in bold.

| Treatment | F-value | p-value |
| --- | --- | --- |
| Plant biomass | 8.81 | **0.004** |

**Table S6. Chi-squared values (χ^2^) of generalized mixed-effects models (GLMM) for the effects of drought, fertilization, and their interaction on soil nematode functional guilds and c-p groups across both seasons (spring and summer 2017).** A random intercept with plots nested within blocks, which were nested within season was added to the model, and we used a binomial error distribution. Due to particularly low densities (see Table S2) no statistical results can be shown for Ca_4_ and Ca_5_ nematodes. ^(*)^ p < 0.1; * p < 0.05; ** p < 0.01

|  |  |  |  |  |  |
| --- | --- | --- | --- | --- | --- |
|  | **Nematode functional categories** | **Drought** | **NPK** | **Drought x NPK** |  |
|  |  | χ^2^ | χ^2^ | χ^2^ |  |
|  | **Ba_1_** | 0.94 | 4.57* | 0.93 |  |
|  | **Ba_2_** | 1.35 | 1.92 | 0.25 |  |
|  | **Ba_4_** | 2.31 | 0.32 | 0.02 |  |
|  | **Fu_2_** | 1.63 | 0.04 | 0.60 |  |
|  | **Fu_3_** | 0.45 | 4.97* | 0.58 |  |
|  | **Fu_4_** | 0.76 | 2.28 | 0.42 |  |
|  | **Om_4_** | 2.23 | 3.80(*) | 0.95 |  |
|  | **Om_5_** | 1.16 | 0.05 | 0.00 |  |
|  | **Ca_4_** | NA | NA | NA |  |
|  | **Ca_5_** | NA | NA | NA |  |
|  | **cp1** | 0.50 | 0.00 | 0.00 |  |
|  | **cp2** | 0.39 | 0.14 | 0.93 |  |
|  | **cp2 PF*** | 6.65** | 1.47 | 2.68 |  |
|  | **cp3** | 0.44 | 2.74(*) | 1.06 |  |
|  | **cp3 PF** | 0.61 | 0.26 | 0.01 |  |
|  | **cp4** | 1.86 | 7.83** | 0.03 |  |
|  | **cp4 PF** | 0.21 | 0.00 | 1.58 |  |
|  | **cp5** | 1.95 | 0.01 | 0.00 |  |
|  | **cp5 PF** | 0.28 | 0.02 | 0.00 |  |
|  |  |  |  |  |  |

*PF-plant feeders

**Table S7. Effects of drought, fertilization, and their interaction on soil pH based on linear mixed-effects models (R² marginal = 0.11, R² conditional = 0.53).** A random intercept with blocks nested within seasons was added to the model and a compound symmetry covariance structure was used to account for repeated measurements within plots. Spring, summer, fall, and winter 2017 were included. Significant results are shown in bold.

| Treatment | F-value | p-value |
| --- | --- | --- |
| Drought  Fertilization  Drought x Fertilization | 0.01  14.11  2.08 | 0.92  **< 0.001**  0.16 |

**Table S8. Effects of soil pH on invertebrate feeding activity (log-scaled) based on linear mixed-effects models (R² marginal = 0.04, R² conditional = 0.41).** A random intercept with blocks nested within seasons was added to the model and a compound symmetry covariance structure was used to account for repeated measurements within plots. Spring, summer, fall, and winter 2017 were included.

| Treatment | F-value | p-value |
| --- | --- | --- |
| pH | 3.40 | 0.07 |

**Table S9. Mean values of soil water content [%) at ambient precipitation (plots without roof constructions).** Shown are means of the non-transformed data (±SD) for each season.

|  | **Spring** | **Summer** | **Fall** | **Winter** |
| --- | --- | --- | --- | --- |
| **Mean** | 14.12 | 12.13 | 11.17 | 19.13 |
| **SD** | 5.86 | 5.12 | 1.72 | 0.28 |

**Table S10. Mean values of soil water content [%] separated by season and the drought treatment.** Shown are means of the non-transformed data (±SD).

|  | **Spring** | | **Summer** | | **Fall** | | **Winter** | |
| --- | --- | --- | --- | --- | --- | --- | --- | --- |
|  | **Drought Treatment** | | | | | | | |
|  | *control* | *reduced* | *control* | *reduced* | *control* | *reduced* | *control* | *reduced* |
|  |  |  |  |  |  |  |  |  |
| **Mean** | 13.22 | 12.58 | 10.20 | 9.32 | 8.61 | 7.70 | 16.47 | 14.36 |
| **SD** | 4.78 | 4.49 | 4.93 | 4.55 | 1.45 | 1.12 | 1.67 | 1.31 |
